# Crystal structure of methyl chloro­formate

**DOI:** 10.1107/S2056989025008369

**Published:** 2025-09-30

**Authors:** Sven Ringelband, Frank Tambornino

**Affiliations:** a Philipps-Universität Marburg, Hans-Meerwein-Str. 4, 35032 Marburg, Germany; Vienna University of Technology, Austria

**Keywords:** crystal structure, Hirshfeld surface analysis, vibrational spectroscopy, methyl chloro­formate

## Abstract

The crystal structure of methyl chloro­formate, C_2_H_3_ClO_2_, was determined by single-crystal X-ray diffraction data at 200 and 100 K. A suitable single crystal was grown in a capillary directly on the goniometer *via* Ostwald ripening around its melting point at 210 K. The ClC(O)OCH_3_ mol­ecule has a staggered conformation and is planar (without H atoms) with point group *C*_S_. In the crystal, methyl chloro­formate features weak Cl⋯O inter­actions forming chains along [100], which again extend into a network *via* inter­molecular C⋯O dipole inter­actions, consistent with Hirshfeld surface analysis. Complementary quantum chemical calculations at the DFT-def2-TZVP/PBE0-D3 level of theory were performed to compare with the experimental Raman data in both the liquid and solid state.

## Abstract 2

In the crystal structure of methyl chloro­formate, the staggered ClC(O)OCH_3_ mol­ecule adopts *C*_S_ symmetry. A phase transition is not observed between 200 and 100 K.

## 1. Chemical context

Methyl chloro­formate, ClC(O)OCH_3_, is a liquid at room temperature with a melting point of 212 K (GESTIS, 2025 ▸). It is widely used in organic chemistry to introduce a meth­oxy­carbonyl functionality to a suitable nucleophile (Chiarucci *et al.*, 2012 ▸). Methyl chloro­formate is well characterized, including its conformational properties by vibrational spectroscopy, microwave spectra (Groner *et al.*, 1990 ▸), and gas phase electron diffraction (O’Gorman *et al.*, 1950 ▸). Herein, we report on its hitherto unknown crystal structure on the basis of diffraction data recorded at 200 and 100 K. Additional quantum chemical calculations were performed and are consistent with the experimental findings and allow for the assignment of the vibrational modes.
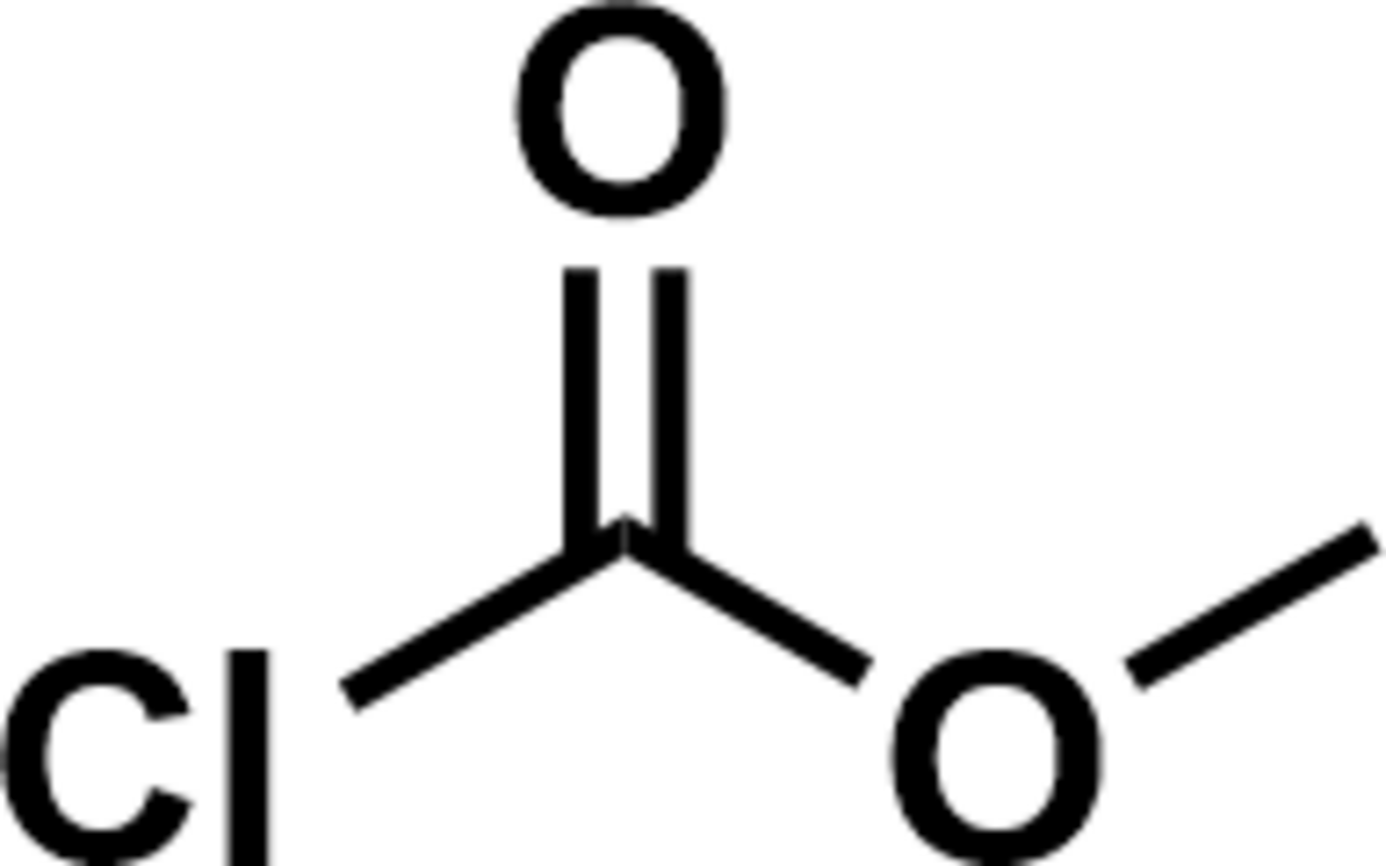


## 2. Structural commentary

**Figure 1:**
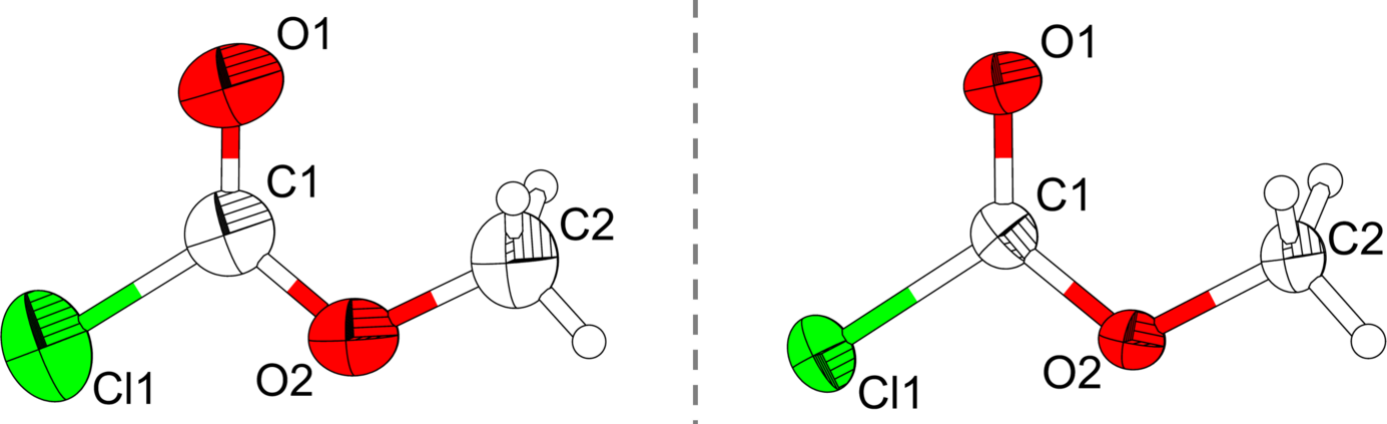
Mol­ecular structure of a methyl chloro­formate mol­ecule in the solid state measured at 200 K (left) and 100 K (right). Atoms are drawn with displacement ellipsoids at the 70% probability level.

**Figure 2:**
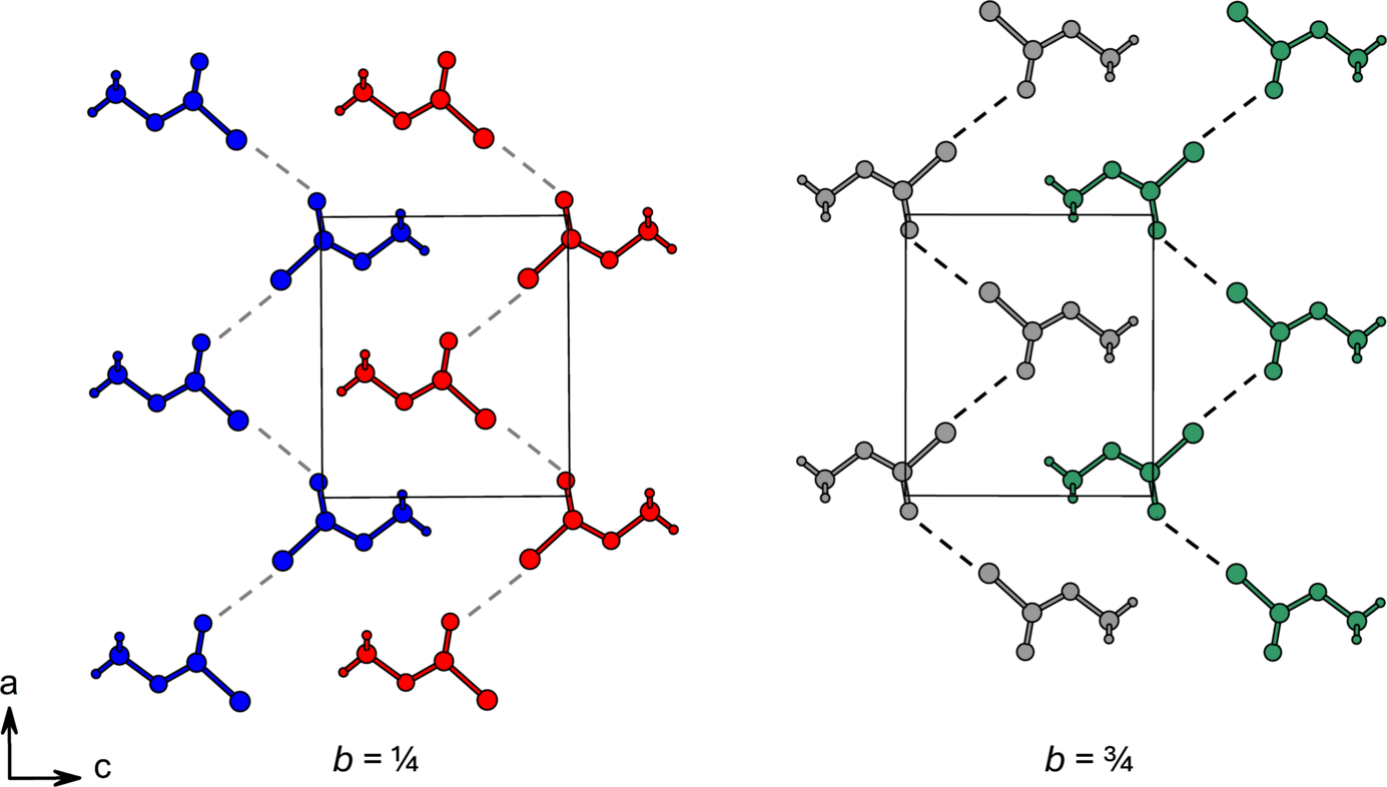
*AB* stacking in the crystal structure of methyl chloro­formate viewed along [010]. Repeating layers: *A* (left) and *B* (right).

Methyl chloro­formate crystallizes in the ortho­rhom­bic crystal system with the centrosymmetric space group *Pnma*. It has been shown that crystals of small mol­ecular compounds can exhibit extensive polymorphism in the solid state, thus undergoing phase transitions at temperatures somewhere below their melting temperatures (Cruz-Cabeza *et al.*, 2015 ▸). However, lowering the temperature starting from 200 K did not lead to a phase transition. The unit cell contains four formula units (*Z* = 4) and one independent mol­ecule in the asymmetric unit (Fig. 1 ▸). In the crystal structure, individual mol­ecules are arranged in an *AB* stacking pattern (Fig. 2 ▸).

In the solid state, ClC(O)OCH_3_ adopts *C*_S_ symmetry with the mol­ecule lying on a mirror plane and thus displaying a torsion angle Cl1—C1—O2—C2 of exactly 180°. Due to the symmetry restrictions of point group *C*_S_, the methyl group is in a staggered orientation with respect to the carbonyl group. Also, the O—CH_3_ moiety is orientated *syn* relative to the C=O bond, with bond lengths similar to those determined by electron diffraction and comparable to those of phosgene (Zaslow *et al.*, 1952 ▸) and dimethyl carbonate (Whitfield, 2023 ▸). A comparison of the structural parameters is given in Table 1 ▸.

## 3. Supra­molecular features

The crystal packing of methyl chloro­formate is dominated by short inter­molecular Cl⋯O contacts with a distance of *d*_Cl⋯O_ = 3.0442 (9) Å, forming infinite chains along [100] (Fig. 2 ▸). Within these chains, the mol­ecules are aligned in a zigzag fashion. However, individual chains are not inter­connected into layers, but instead form a framework through weak inter­molecular dipole inter­actions between the carbonyl oxygen atom and the central carbon atom of two parallel mol­ecules along [010].

**Figure 3:**
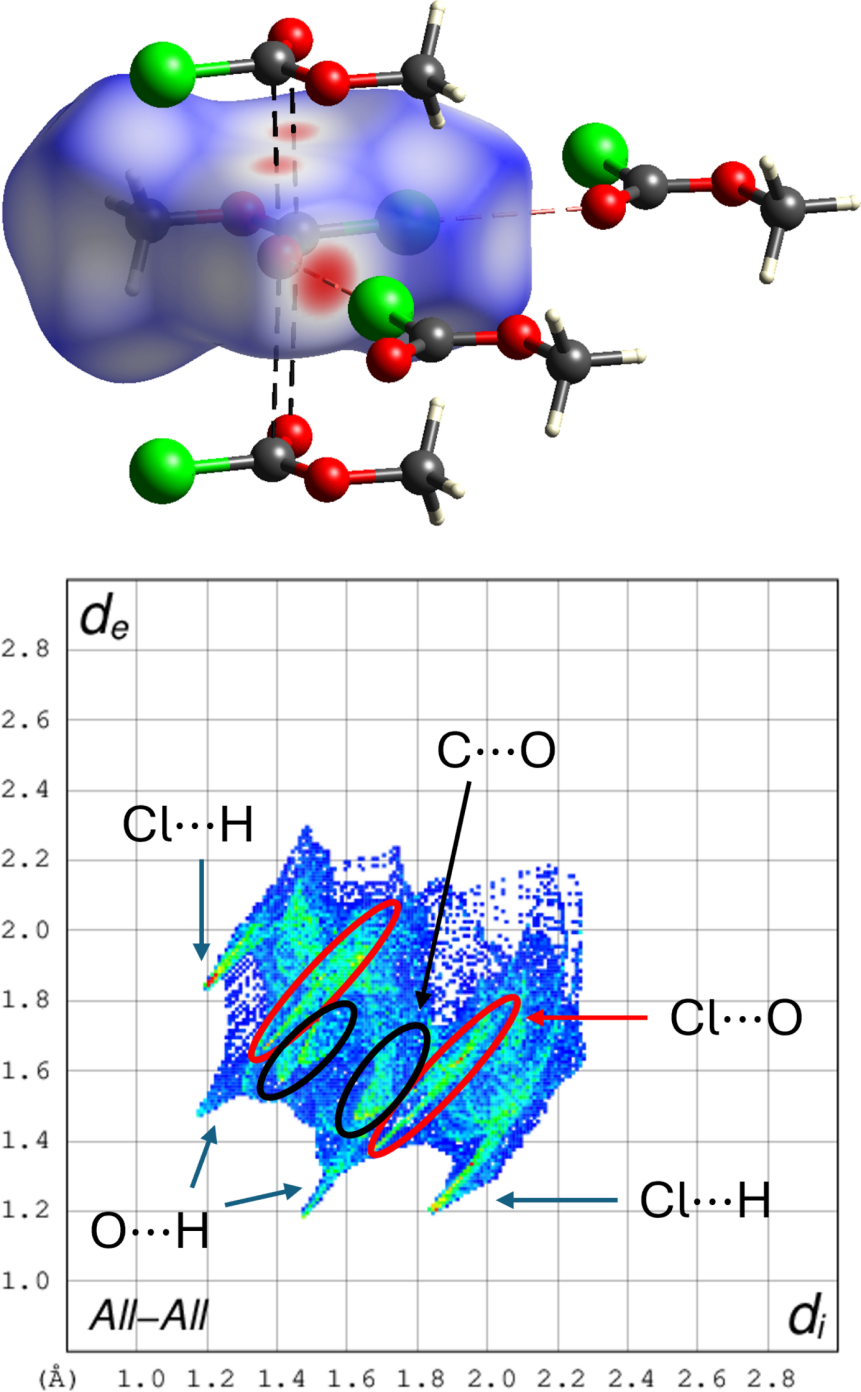
Hirshfeld surface (top) and fingerprint plot (bottom) mapped for methyl chloro­formate on basis of 100 K data. Red areas indicate short contacts shorter than the sum of the van der Waals radii. Color code: C gray, O red, Cl green, H white.

A Hirshfeld surface analysis was performed using *CrystalExplorer* (Spackman *et al.*, 2021 ▸) to qu­antify and visualize the inter­molecular inter­actions in the crystal structure of the title compound. The Hirshfeld surface was mapped with the *d*_norm_ function (Fig. 3 ▸), highlighting attractive inter­actions shorter than the sum of the van der Waals radii in red and equal or longer contacts in white and blue, respectively. Akin to the observations above, relevant contributions arise from Cl⋯O and C⋯O short contacts accounting for only 12.6% and 7.6% to the overall Hirshfeld plot. Other major contributions include Cl—H (35.0%) and O—H (27.4%) inter­molecular contacts. However, those are equal to or longer than the sum of their van der Waals radii.

## 4. Vibrational spectroscopy

**Figure 4:**
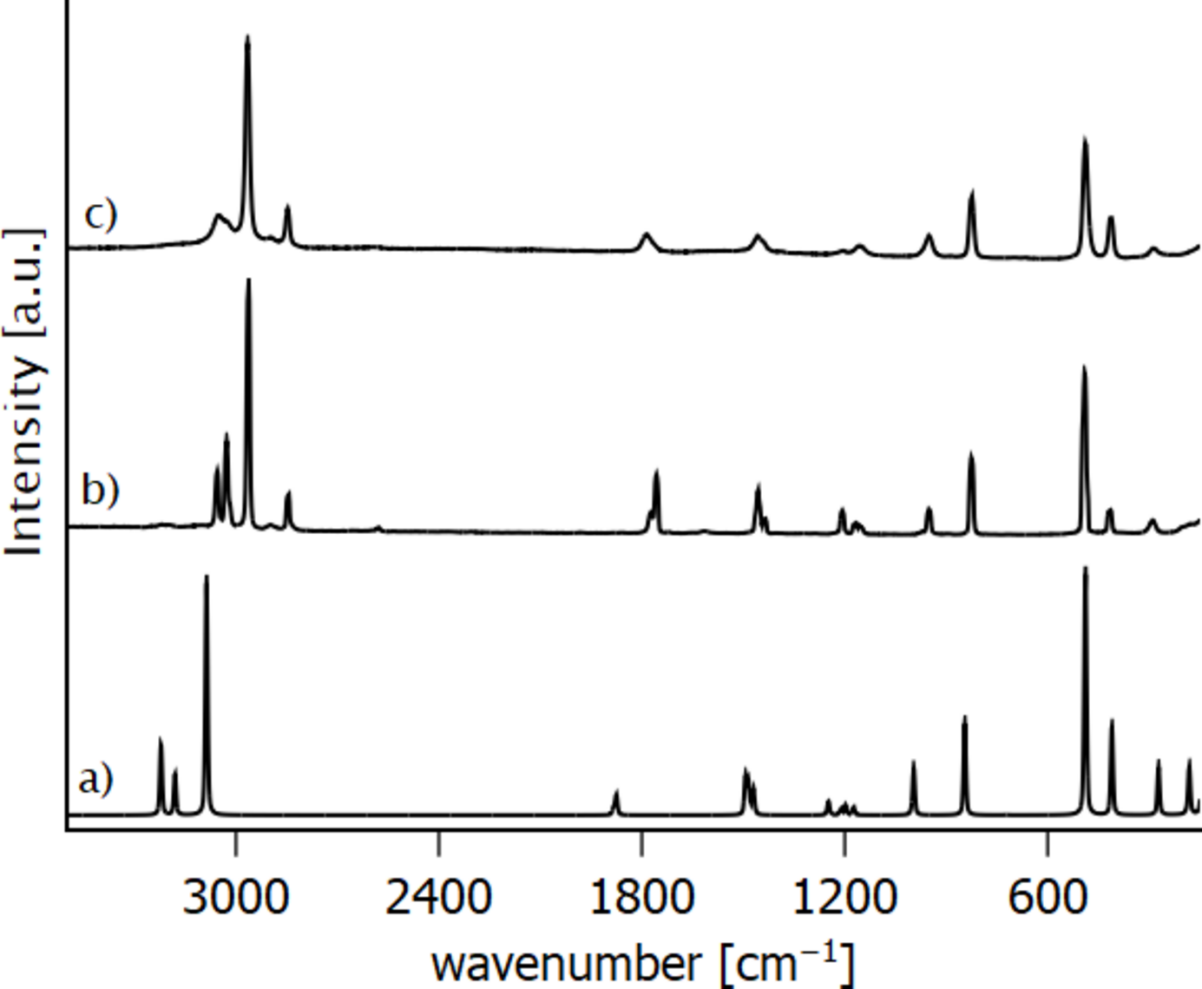
Calculated (*a*) and recorded Raman spectra of methyl chloro­formate (*b*) at room temperature (liquid) and (*c*) at 153 K (crystalline). Calculated Raman spectra of solid ClC(O)OCH_3_ at the DFT-TZVP/PBE0 level of theory.

Experimental Raman spectra of liquid and solid methyl chloro­formate were recorded at room temperature and at 153 K, confirming the mol­ecular structure as determined by X-ray analysis. The experimental spectra are confirmed by quantum chemical calculations at the DFT-def2-TZVP/PBE0-D3 level of theory (Weigend & Ahlrichs, 2005 ▸; Karttunen *et al.*, 2015 ▸; Dovesi *et al.*, 2018 ▸; Zicovich *et al.*, 2004 ▸; Pascale *et al.*, 2004 ▸; Maschio *et al.*, 2013 ▸; Grimme *et al.*, 2010 ▸) on the basis of the crystal structure of methyl chloro­formate. The recorded and calculated spectra are in good agreement, as shown in Fig. 4 ▸. The vibrational frequencies have been calculated within the harmonic approximation and therefore are overestimated, especially at higher wavenumbers. Nevertheless, the vibrational band at 2845 cm^−1^ is not reproduced in the calculated spectrum. A comparison of calculated and observed vibrational bands is given in Table 2 ▸.

## 5. Synthesis and crystallization

Methyl chloro­formate (Fisher Scientific GmbH, 99%) was used as received. Its purity was checked with vibrational IR and Raman spectroscopy. A borosilicate glass capillary (0.3 mm, Hilgenberg) was filled with a small amount of methyl chloro­formate and flame-sealed at ambient pressure. The capillary was mounted onto the goniometer of the diffractometer and shock-cooled at 100 K using an open-flow cryostat to obtain a polycrystalline sample. The sample was incrementally heated at a rate of 180 K min^−1^ until partial melting was observed at around 213 K. Subsequently, a suitable single crystal was grown through Ostwald ripening between 200 and 213 K in three cycles. Full datasets were collected at 200 and 100 K and reflections of the strongest scattering individuum integrated.

## 6. Refinement

Crystal data, data collection and structure refinement details are summarized in Table 3 ▸. The positions of the hydrogen atoms were obtained through difference-Fourier synthesis in both datasets.

## Supplementary Material

Crystal structure: contains datablock(s) global, srx03, srx02. DOI: 10.1107/S2056989025008369/wm5765sup1.cif

Structure factors: contains datablock(s) srx03. DOI: 10.1107/S2056989025008369/wm5765srx03sup2.hkl

Supporting information file. DOI: 10.1107/S2056989025008369/wm5765srx03sup4.cml

Structure factors: contains datablock(s) srx02. DOI: 10.1107/S2056989025008369/wm5765srx02sup3.hkl

Supporting information file. DOI: 10.1107/S2056989025008369/wm5765srx02sup5.cml

CCDC references: 2490420, 2490419

Additional supporting information:  crystallographic information; 3D view; checkCIF report

## Figures and Tables

**Table 1:** Comparison of selected inter­atomic distances (Å, °) of methyl chloro­formate (X-ray, this work), methyl chloro­formate (electron diffraction; O’Gorman *et al.*, 1950 ▸), phosgene (Zaslow *et al.*, 1952 ▸) and dimethyl carbonate (Whitfield, 2023 ▸)

	X-ray 200 K	X-ray 100 K	Electron diffraction	Phosgene	Dimethyl carbonate 82 K
C=O	1.186 (2)	1.195 (2)	1.19 (3)	1.15 (2)	1.219 (2)
C—Cl	1.7504 (13)	1.7502 (13)	1.75 (2)	1.74 (2)^*^	–
(CO)—O	1.306 (2)	1.309 (2)	1.36 (4)	–	1.337 (2)^*^
O—CH_3_	1.456 (2)	1.462 (2)	1.47 (4)	–	1.456 (2)^*^
Cl—C=O	122.46 (10)	122.47 (10)	–	124(1.5)^*^	–
Cl—C—O	108.61 (9)	108.75 (9)	112 (3)	–	–
O=C—O	128.93 (11)	128.78 (11)	126 (4)	–	125.58 (11)^*^

^*^Mean values.

**Table 2:** Vibrational band positions (cm^−1^) and band assignments of the Raman spectra of liquid and solid methyl chloro­formate based on the DFT calculation. Symmetry modes are given in parentheses.

*ν*_calc._	*v*_obs._(liquid)	*v*_obs._(solid)	**Assignment**	*ν*_calc._	*v*_obs._(liquid)	*v*_obs._(solid)	**Assignment**
182 (*B*_2g_)	–	195	out-of-plane *δ*(C—O—C)	1248 (*A*_g_)	1206	1206	*ν*(C—O)+*δ*(CH_3_)
191 (*B*_1g_)				1253 (*B*_3g_)			
272 (*B*_3g_)	281	283	in-plane *δ*(C—O—C)	1470 (*B*_3g_)	1457	1435	*δ*_s_(CH_3_)
272 (*A*_g_)				1474 (*A*_g_)			
409 (*B*_3g_)	407	411	in-plane *δ*(C—O—C—Cl)	1485 (*A*_g_)		1452	*δ*_s_(CH_3_)
410 (*A*_g_)				1486 (*B*_3g_)			
488 (*A*_g_)	485	481	*ν*(C—Cl)	1491 (*B*_1g_)		1459	*δ*_as_(CH_3_)
490 (*B*_3g_)		486		1494 (*B*_2g_)			
719 (*B*_1g_)	–	–	out-of-plane *ν*_as_(O—C—O) wagging	1875 (*A*_g_)	1788	1756	*ν*(C=O)
723 (*B*_2g_)	–	–		1884 (*B*_3g_)		1778	
845 (*A*_g_)	823	824	*δ*(C—O—C—Cl) scissoring	3087 (*A*_g_)	2967	2964	*ν*_s_(CH_3_)
852 (*B*_3g_)				3088 (*B*_3g_)			
996 (*A*_g_)	950	950	*ν*(O—CH_3_)	3180 (*B*_2g_)	3026	3029	*ν*_as_(CH_3_)
1004 (*B*_3g_)		975		3180 (*B*_1g_)			
1174 (*B*_1g_)	1155	1148	*δ*(CH_3_) rocking	3222 (*A*_g_)	3053	3056	*ν*_as_(CH_3_)
1175 (*B*_2g_)				3222 (*B*_3g_)			
1198 (*A*_g_)		1155	*δ*(CH_3_) rocking				
1212 (*B*_3g_)		1168					

Notes: *ν* = stretching vibration, *δ* = bending vibration, *s* = symmetric, *as* = asymmetric, – = not observed.

**Table 3:** Experimental details

	100 K	200 K
Crystal data		
Chemical formula	C_2_H_3_ClO_2_	C_2_H_3_ClO_2_
*M*_r_	94.49	94.49
Crystal system, space group	Orthorhombic, *Pnma*	Orthorhombic, *Pnma*
*a*, *b*, *c* (Å)	8.4404 (5), 6.2019 (4), 7.4314 (4)	8.526 (2), 6.3454 (15), 7.4619 (16)
*V* (Å^3^)	389.01 (4)	403.67 (16)
*Z*	4	4
Radiation type	Cu *K*α	Cu *K*α
μ (mm^−1^)	7.23	6.97
Crystal size (mm)	0.30 × 0.05 (radius)	0.30 × 0.15 (radius)

Data collection		
Diffractometer	Stoe Stadivari	Stoe Stadivari
Absorption correction	Multi-scan (*LANA*; Koziskova *et al.*, 2016 ▸)	Multi-scan (*LANA*; Koziskova *et al.*, 2016 ▸)
*T*_min_, *T*_max_	0.161, 0.337	0.231, 0.512
No. of measured, independent and observed [*I* > 2σ(*I*)] reflections	8421, 432, 415	10088, 453, 432
*R*_int_	0.018	0.022
(sin θ/λ)_max_ (Å^−1^)	0.627	0.625

Refinement		
*R*[*F*^2^ > 2σ(*F*^2^)], *wR*(*F*^2^), *S*	0.018, 0.049, 1.08	0.022, 0.064, 1.11
No. of reflections	432	453
No. of parameters	38	39
H-atom treatment	All H-atom parameters refined	All H-atom parameters refined
Δρ_max_, Δρ_min_ (e Å^−3^)	0.18, −0.17	0.18, −0.16

Computer programs: *X-AREA* (Stoe, 2022 ▸), *SHELXT* (Sheldrick, 2015*a* ▸), *SHELXL* (Sheldrick, 2015*b* ▸) and *OLEX2* (Dolomanov *et al.*, 2009 ▸).

## supplementary crystallographic information

### Methyl chloroformate (srx03) Crystal data

**Table:**

C_2_H_3_ClO_2_	*D*_x_ = 1.613 Mg m^−^^3^
*M**_r_* = 94.49	Cu *K*α radiation, λ = 1.54186 Å
Orthorhombic, *P**n**m**a*	Cell parameters from 30155 reflections
*a* = 8.4404 (5) Å	θ = 5.3–75.7°
*b* = 6.2019 (4) Å	µ = 7.23 mm^−^^1^
*c* = 7.4314 (4) Å	*T* = 100 K
*V* = 389.01 (4) Å^3^	Block, clear colourless
*Z* = 4	0.30 × 0.15 × 0.15 × 0.05 (radius) mm
*F*(000) = 192	

### Methyl chloroformate (srx03) Data collection

**Table:**

Stoe Stadivari diffractometer	432 independent reflections
Radiation source: microfocus sealed X-ray tube, XENOCS GENIX 3D CU HIGH FLUX	415 reflections with *I* > 2σ(*I*)
Graded multilayer mirror monochromator	*R*_int_ = 0.018
Detector resolution: 5.81 pixels mm^-1^	θ_max_ = 75.4°, θ_min_ = 8.0°
ω and φ scans	*h* = −8→10
Absorption correction: multi-scan (*LANA*; Koziskova *et al.*, 2016)	*k* = −7→7
*T*_min_ = 0.161, *T*_max_ = 0.337	*l* = −9→9
8421 measured reflections	

### Methyl chloroformate (srx03) Refinement

**Table:**

Refinement on *F*^2^	Primary atom site location: dual
Least-squares matrix: full	Hydrogen site location: difference Fourier map
*R*[*F*^2^ > 2σ(*F*^2^)] = 0.018	All H-atom parameters refined
*wR*(*F*^2^) = 0.049	*w* = 1/[σ^2^(*F*_o_^2^) + (0.0349*P*)^2^ + 0.0604*P*] where *P* = (*F*_o_^2^ + 2*F*_c_^2^)/3
*S* = 1.08	(Δ/σ)_max_ = 0.001
432 reflections	Δρ_max_ = 0.18 e Å^−^^3^
38 parameters	Δρ_min_ = −0.17 e Å^−^^3^
0 restraints	

### Methyl chloroformate (srx03) Special details

**Table:**

Geometry. All esds (except the esd in the dihedral angle between two l.s. planes) are estimated using the full covariance matrix. The cell esds are taken into account individually in the estimation of esds in distances, angles and torsion angles; correlations between esds in cell parameters are only used when they are defined by crystal symmetry. An approximate (isotropic) treatment of cell esds is used for estimating esds involving l.s. planes.

### Methyl chloroformate (srx03) Fractional atomic coordinates and isotropic or equivalent isotropic displacement parameters (Å^2^)

**Table:**

	*x*	*y*	*z*	*U*_iso_*/*U*_eq_	
Cl1	0.72372 (4)	0.750000	0.33814 (4)	0.02357 (16)	
O2	0.65970 (11)	0.750000	0.66663 (10)	0.0201 (2)	
O1	0.44492 (10)	0.750000	0.48536 (12)	0.0235 (2)	
C1	0.58441 (15)	0.750000	0.51257 (16)	0.0179 (3)	
C2	0.55644 (18)	0.750000	0.82457 (17)	0.0238 (3)	
H2A	0.4907 (18)	0.622 (2)	0.8235 (14)	0.033 (3)*	
H2B	0.622 (2)	0.750000	0.919 (3)	0.037 (5)*	

### Methyl chloroformate (srx03) Atomic displacement parameters (Å^2^)

**Table:**

	*U*^11^	*U*^22^	*U*^33^	*U*^12^	*U*^13^	*U*^23^
Cl1	0.0234 (2)	0.0267 (2)	0.0206 (2)	0.000	0.00467 (10)	0.000
O2	0.0148 (5)	0.0269 (5)	0.0186 (5)	0.000	0.0001 (4)	0.000
O1	0.0174 (5)	0.0283 (5)	0.0246 (5)	0.000	−0.0034 (4)	0.000
C1	0.0190 (6)	0.0154 (6)	0.0193 (6)	0.000	0.0008 (5)	0.000
C2	0.0213 (7)	0.0327 (8)	0.0174 (6)	0.000	0.0014 (5)	0.000

### Methyl chloroformate (srx03) Geometric parameters (Å, º)

**Table:**

Cl1—C1	1.7502 (13)	C2—H2A	0.968 (14)
O2—C1	1.3094 (15)	C2—H2A^i^	0.968 (14)
O2—C2	1.4619 (15)	C2—H2B	0.90 (2)
O1—C1	1.1945 (15)		
			
C1—O2—C2	114.37 (10)	O2—C2—H2A	109.6 (7)
O2—C1—Cl1	108.75 (9)	O2—C2—H2B	105.1 (12)
O1—C1—Cl1	122.47 (10)	H2A—C2—H2A^i^	110.1 (17)
O1—C1—O2	128.78 (11)	H2A—C2—H2B	111.2 (9)
O2—C2—H2A^i^	109.6 (7)	H2B—C2—H2A^i^	111.2 (9)
			
C2—O2—C1—Cl1	180.000 (1)	C2—O2—C1—O1	0.000 (1)

Symmetry code: (i) *x*, −*y*+3/2, *z*.

### Methyl chloroformate (srx02) Crystal data

**Table:**

C_2_H_3_ClO_2_	*D*_x_ = 1.555 Mg m^−^^3^
*M**_r_* = 94.49	Cu *K*α radiation, λ = 1.54186 Å
Orthorhombic, *P**n**m**a*	Cell parameters from 21811 reflections
*a* = 8.526 (2) Å	θ = 5.9–75.5°
*b* = 6.3454 (15) Å	µ = 6.97 mm^−^^1^
*c* = 7.4619 (16) Å	*T* = 200 K
*V* = 403.67 (16) Å^3^	Block, clear colourless
*Z* = 4	0.30 × 0.15 × 0.15 × 0.15 (radius) mm
*F*(000) = 192	

### Methyl chloroformate (srx02) Data collection

**Table:**

Stoe Stadivari diffractometer	453 independent reflections
Radiation source: microfocus sealed X-ray tube, XENOCS GENIX 3D CU HIGH FLUX	432 reflections with *I* > 2σ(*I*)
Graded multilayer mirror monochromator	*R*_int_ = 0.022
Detector resolution: 5.81 pixels mm^-1^	θ_max_ = 74.6°, θ_min_ = 7.9°
ω and φ scans	*h* = −8→10
Absorption correction: multi-scan (*LANA*; Koziskova *et al.*, 2016)	*k* = −7→7
*T*_min_ = 0.231, *T*_max_ = 0.512	*l* = −9→9
10088 measured reflections	

### Methyl chloroformate (srx02) Refinement

**Table:**

Refinement on *F*^2^	Hydrogen site location: difference Fourier map
Least-squares matrix: full	All H-atom parameters refined
*R*[*F*^2^ > 2σ(*F*^2^)] = 0.022	*w* = 1/[σ^2^(*F*_o_^2^) + (0.0492*P*)^2^ + 0.0148*P*] where *P* = (*F*_o_^2^ + 2*F*_c_^2^)/3
*wR*(*F*^2^) = 0.064	(Δ/σ)_max_ < 0.001
*S* = 1.11	Δρ_max_ = 0.18 e Å^−^^3^
453 reflections	Δρ_min_ = −0.16 e Å^−^^3^
39 parameters	Extinction correction: SHELXL-2018/3 (Sheldrick 2015b), Fc^*^=kFc[1+0.001xFc^2^λ^3^/sin(2θ)]^-1/4^
0 restraints	Extinction coefficient: 0.0081 (16)
Primary atom site location: dual	

### Methyl chloroformate (srx02) Special details

**Table:**

Geometry. All esds (except the esd in the dihedral angle between two l.s. planes) are estimated using the full covariance matrix. The cell esds are taken into account individually in the estimation of esds in distances, angles and torsion angles; correlations between esds in cell parameters are only used when they are defined by crystal symmetry. An approximate (isotropic) treatment of cell esds is used for estimating esds involving l.s. planes.

### Methyl chloroformate (srx02) Fractional atomic coordinates and isotropic or equivalent isotropic displacement parameters (Å^2^)

**Table:**

	*x*	*y*	*z*	*U*_iso_*/*U*_eq_	
Cl1	0.27937 (4)	0.750000	0.33697 (4)	0.0485 (2)	
O2	0.34065 (11)	0.750000	0.66399 (10)	0.0382 (3)	
O1	0.55347 (11)	0.750000	0.48601 (12)	0.0472 (3)	
C1	0.41624 (15)	0.750000	0.51182 (15)	0.0333 (3)	
C2	0.44070 (19)	0.750000	0.82207 (18)	0.0465 (4)	
H2A	0.377 (3)	0.750000	0.917 (3)	0.068 (6)*	
H2B	0.505 (2)	0.627 (2)	0.8228 (15)	0.064 (4)*	

### Methyl chloroformate (srx02) Atomic displacement parameters (Å^2^)

**Table:**

	*U*^11^	*U*^22^	*U*^33^	*U*^12^	*U*^13^	*U*^23^
Cl1	0.0511 (3)	0.0547 (3)	0.0396 (3)	0.000	−0.01092 (11)	0.000
O2	0.0267 (5)	0.0527 (6)	0.0352 (5)	0.000	0.0021 (3)	0.000
O1	0.0336 (5)	0.0621 (7)	0.0458 (6)	0.000	0.0086 (4)	0.000
C1	0.0334 (6)	0.0313 (6)	0.0353 (7)	0.000	0.0015 (5)	0.000
C2	0.0412 (8)	0.0649 (9)	0.0333 (7)	0.000	−0.0021 (5)	0.000

### Methyl chloroformate (srx02) Geometric parameters (Å, º)

**Table:**

Cl1—C1	1.7504 (13)	C2—H2A	0.89 (2)
O2—C1	1.3057 (15)	C2—H2B^i^	0.953 (17)
O2—C2	1.4557 (16)	C2—H2B	0.953 (17)
O1—C1	1.1856 (16)		
			
C1—O2—C2	114.55 (11)	O2—C2—H2B	109.9 (8)
O2—C1—Cl1	108.61 (9)	O2—C2—H2B^i^	109.9 (8)
O1—C1—Cl1	122.46 (10)	H2A—C2—H2B	110.0 (10)
O1—C1—O2	128.93 (11)	H2A—C2—H2B^i^	110.0 (10)
O2—C2—H2A	106.8 (14)	H2B—C2—H2B^i^	110 (2)
			
C2—O2—C1—Cl1	180.000 (1)	C2—O2—C1—O1	0.000 (1)

Symmetry code: (i) *x*, −*y*+3/2, *z*.
